# Traffic Noise Assessment in Urban Puducherry, South India

**DOI:** 10.7759/cureus.51975

**Published:** 2024-01-09

**Authors:** Debajyoti Bhattacharya, James Devasia, Subitha Lakshminarayanan, Mahalakshmy Thulasingam

**Affiliations:** 1 Department of Preventive and Social Medicine, Jawaharlal Institute of Postgraduate Medical Education and Research, Puducherry, IND

**Keywords:** noise pollution level, traffic noise index, noise climate, noise exposure index, temporal variations, noise pollution

## Abstract

Background

Noise pollution is an emerging global problem that can affect people's well-being and mental and physical health. In India, six percent of people suffer hearing loss, and prolonged exposure leads to irreversible noise-induced hearing loss.

Objective

To assess the noise levels at selected residential, commercial, industrial, silence zones, traffic junctions, and related noise indices in urban Puducherry and compare them with Central Pollution Control Board (CPCB) standards.

Methods

The study was conducted using a cross-sectional noise survey based on the 2015 study sites in urban Puducherry using a sound level meter, analyzed the results with limits set by the CPCB standards, and calculated the various noise indices.

Results

In urban Puducherry, the noise level showing silence zones is more hazardous than industrial, residential, commercial, and traffic junctions. Out of the 36 sites surveyed, 33 locations are above the prescribed daytime limit by CPCB.

Conclusions

The noise assessment at selected sites in urban Puducherry shows that around 92% of study sites are well above the daytime standards of CPCB, highlighting an urgent need to curb noise levels. The findings revealed that increased noise at study sites could be due to the increased number of vehicles and transportation systems.

## Introduction

The World Health Organization (WHO) stated noise pollution is the third most harmful pollutant after air and water, and if it surpasses 75 dB, it can affect both auditory and non-auditory health. Nearly 10% of the global population is exposed to noise pollution [1], and over 2.5 billion people are estimated to have hearing loss by 2050 [2]. The burden of disease analysis done in developed countries shows one out of 100 premature deaths [3] and an increased risk for ischemic heart disease (IHD) and stroke among women exposed to road traffic noise from multiple sources [4].

Several studies performed in the western, central, and northern parts of India have revealed that noise levels in different zones at various time slots exceed the standards prescribed by the Central Pollution Control Board (CPCB) of the Government of India [5-7]. A study done in Varanasi explored the dynamics of traffic noise and its characteristics [8]. In south India, studies done in various cities in Tamil Nadu show noise levels exceeding the limit, especially in silence and residential zones [9-12]. This study aims to assess the noise levels (LAeq) at various hours of the day among commercial, industrial, residential, and traffic junctions in urban Puducherry and compare them with the CPCB standard along with the noise exposure index (NEI), noise climate (NC), noise pollution level (Lnp), and traffic noise index (TNI).

## Materials and methods

Study design

A cross-sectional analytical study was conducted from July to August 2021.

Study area

**Figure 1:**
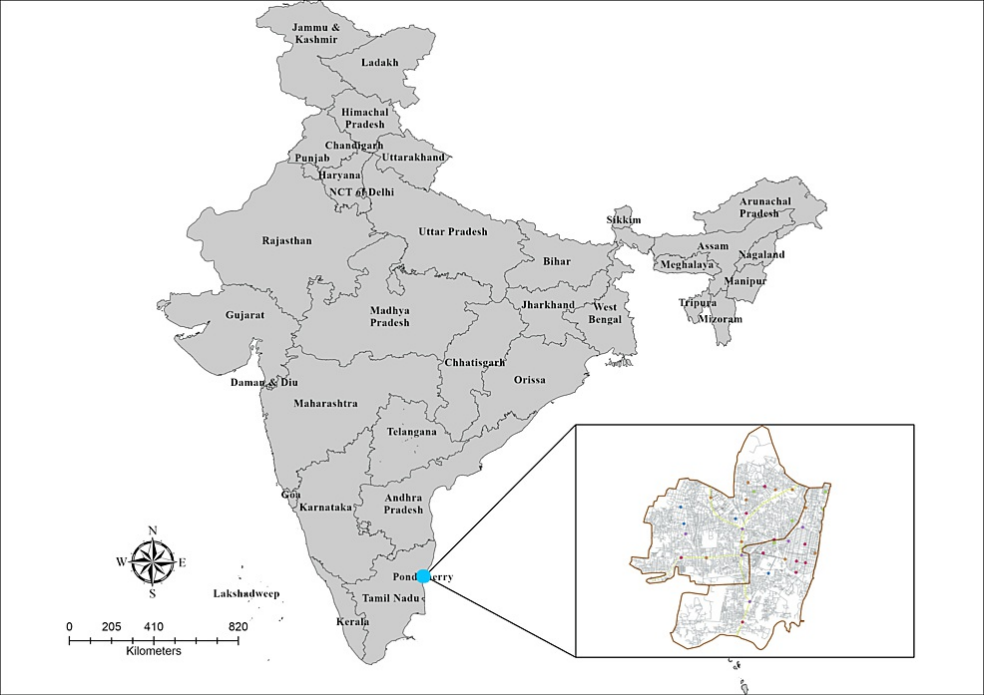
Map of India and location of Puducherry The location of study sites in Puducherry is shown in the inset. Maps were generated by the authors using ArcGIS Pro 2.7 software (https://pro.arcgis.com/en/). It's a creation by the authors, not any copyright from previous studies.

The capital of the Union Territory (UT), Puducherry, is located between 11° 55′ 52.44′′ N and 79° 50′ 6.95′′ E. Urban Puducherry has a total population of 657,209, incorporating two important municipalities, Puducherry and Oulgaret [13]. This study covers a total area of 41.92 km², out of which 24.10 km² is in Oulgaret Municipality. About 69.13% of the Puducherry population resides in urban areas, with the population density in urban Puducherry being 3232 per sq. km. [14]. Figure 1 shows the location of the study sites.

Sample size and sampling technique

Grid sampling was done based on the previous study, with a size of 500 m x 500 m. We chose the exact study locations, sampling strategy, inclusion and exclusion criteria, and operational definitions of the study zones from the 2015 study [15]. We have carefully selected 24 sites for every 15 intersection points in the grid system, similar to the previous study. Additionally, we have employed a random selection method to pick 12 study locations with high noise levels, such as commercial, industrial, and traffic intersections. This study aims to examine the fluctuations in noise decibel levels from the previous study as well as to calculate various noise indices.

The grid size was chosen based on the law of physics: when you double the distance from the source, the sound pressure level will reduce by six decibels, as depicted in Table 1.

**Table 1:** Sound pressure levels change with distance Noise is measured in decibels (dB), and distance is measured in feet (ft)

Distance from sound source	Attenuated Noise decibel (dB)
Noise at point	90dB
Reduction of Noise (100 ft)	84dB
Reduction of Noise (200 ft)	78dB
Reduction of Noise (400 ft)	72dB
Reduction of Noise (800 ft)	66dB
Reduction of Noise (1600 ft)	60db

1600 ft is 487.68m, and we rounded up to 500m, so the assumption was in an ideal situation. 90 dB will reduce to an acceptable level at a distance of 500m, so we chose a 500m grid size. Hence, sites that are within 500m of each other were excluded.

Monitoring equipment

Cygnet Datalogging Sound Level Meter (SLM) 2511, Baseline Technologies, New Delhi, India (https://www.baselinetechno.com/). The Sound Level Meter consists of a high-quality electret omnidirectional microphone, 30-134 dB (A) measurement, and Type 1 accuracy as per IS9779.

Garmin Oregon 550 Digital Global Positioning System (DGPS), Garmin Ltd., Kansas, United States (https://www.garmin.co.in). The latitude and longitude of the site were collected with an accuracy of 10 ft to 16 ft (3 m to 5 m) with 95% typical.

Noise standards for industrial, commercial, residential, and silence zones in other developed countries are illustrated in Table 2. The Indian (CPCB) standard noise level in the industrial zone is 5-15 decibels higher, 5-10 decibels higher in commercial and residential zones, and 5 decibels higher in the silence zone when compared to other developed countries' standards.

**Table 2:** Noise standards of some given countries US: United States, EPA: Environmental Protection Agency, WHO: World Health Organization, EC: European Commission, CPCB standards, India:  Daytime is reckoned between 6 a.m. and 10 p.m. Nighttime is reckoned between 10 p.m. and 6 a.m.

Country	Industrial Area Days/Night	Commercial Area Days/Night	Residential Area Days/Night	Silence Area Days/Night
Australia	65/55	55/45	45/35	45/35
India	75/70	65/55	55/45	50/40
Japan	60/50	60/50	50/40	45/35
U.S. (EPA)	70/60	60/50	55/45	45/35
WHO & EC	65	55	55/45	45/35

Monitoring parameters and noise indices

Hourly A-weighted equivalent sound level (LAeq) was recorded in one-second sampling as per the CPCB specifications for measuring ambient sound pressure level. Noise indices were calculated using the following formula:

CPCB % Calculation=(LAeq )/CPCB day time limit for particular zone)×100

\text{CPCB \% Calculation} = \left(\frac{L_{Aeq}}{\text{CPCB day time limit for Particular Zone}}\right) \times 100

Noise exposure index (NEI) = LAeq/Exposure noise limit

\text{Noise Exposure Index (NEI)} = \frac{L_{Aeq}}{\text{Exposure Noise Limit}} [16]

Noise climate (NC)= (L10-L90) dB(A)

\text{Noise Climate (NC)} = (L_{10} - L_{90}) \, \text{dB(A)} [8]

Traffic noise index (TNI)=(L90+4×NC-30) dB(A)

\text{Traffic Noise Index (TNI)} = (L_{90} + 4 \times \text{NC} - 30) \, \text{dB(A)}** ** [8,17]

Noise pollution level (Lnp)=(L50 + (NC)² /60+NC) dB(A)

\text{Noise Pollution Level (Lnp)} = \left(L_{50} + \frac{(\text{NC})^2}{60} + \text{NC}\right) \, \text{dB(A)} [17] 

Noise monitoring and retrieval: Noise intensity was recorded at three different time slots as per the 2015 study: morning (T1) 8:00 a.m. to 9 a.m., noon (T2) 12:00 p.m. to 1:00 p.m., and evening (T3) 6:00 p.m. to 7:00 p.m. SLM 2511 was calibrated before each measurement. Data from SLM 2511 was retrieved using DLO3 software from Baseline Technologies, New Delhi, India (https://www.baselinetechno.com/).

Ethical approval was obtained from the Jawaharlal Institute of Postgraduate Medical Education and Research (JIPMER) Institute Ethics Committee (IEC approval number: JIP/IEC/2021/0147) and Post Graduate Research Monitoring Committee (PGRMC).

## Results

**Figure 2:**
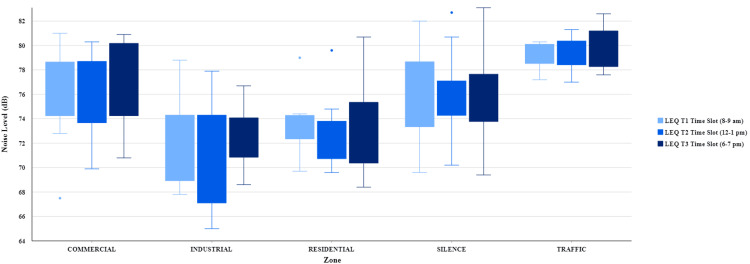
Distribution of noise level (LAeq) for T1, T2, and T3 time slots in commercial, industrial, silence, residential zones, and traffic intersections in selected sites of urban Puducherry, South India, 2021 The authors generated the plot using ArcGIS Pro 2.7 software (https://pro.arcgis.com/en/). LEQ is similar to LAeq.

The distribution of equivalent weighted s (time-averaged): noise level is shown in Figure 2, and Tables 3, 4 illustrate the noise level measured in the selected commercial, silence, industrial, residential, zones, and traffic intersections and their comparison with CPCB standards for T1, T2, and T3 time slots.

**Table 3:** Noise monitoring data and comparison of noise levels with CPCB and NEI for T1, T2, and T3 time slots in commercial and silence zones study sites in urban Puducherry, South India, 2021 LAeq: hourly A-weighted equivalent sound level. Central Pollution Control Board (CPCB): %. NEI: Noise Exposure Index.

Study Sites	LAeq	CPCB %	LAeq	CPCB %	LAeq	CPCB %	NEI		
	T1		T2		T3		T1	T2	T3
Commercial Zone 1	77.5	119.2	77.1	118.6	79.5	122.3	0.7	0.7	0.8
Commercial Zone 2	77.9	119.8	77.2	118.8	77.4	119.1	0.7	0.7	0.7
Commercial Zone 3	78.9	121.4	80.0	123.1	80.9	124.5	0.8	0.8	0.8
Commercial Zone 4	67.5	103.8	69.9	107.5	70.8	108.9	0.6	0.7	0.7
Commercial Zone 5	77.4	119.1	77.2	118.8	75.0	115.4	0.7	0.7	0.7
Commercial Zone 6	73.8	113.5	73.5	113.1	72.4	111.4	0.7	0.7	0.7
Commercial Zone 7	75.6	116.3	74.2	114.2	76.7	118.0	0.7	0.7	0.7
Commercial Zone 8	72.8	112.0	73.0	112.3	74.0	113.8	0.7	0.7	0.7
Commercial Zone 9	81.0	124.6	80.3	123.5	80.8	124.3	0.8	0.8	0.8
Commercial Zone 10	80.8	124.3	79.2	121.8	80.4	123.7	0.8	0.8	0.8
Silence Zone 1	78.9	157.8	74.8	149.6	75.2	150.4	0.8	0.7	0.7
Silence Zone 2	76.4	152.8	75.5	151.0	73.5	147.0	0.7	0.7	0.7
Silence Zone 3	69.6	139.2	70.2	140.4	71.3	142.6	0.7	0.7	0.7
Silence Zone 4	82.0	164.0	82.7	165.4	83.1	166.2	0.8	0.8	0.8
Silence Zone 5	78.0	156.0	77.4	154.8	78.1	156.2	0.7	0.7	0.7
Silence Zone 6	73.3	146.6	74.1	148.2	74.8	149.6	0.7	0.7	0.7
Silence Zone 7	73.5	147.0	70.7	141.4	69.4	138.8	0.7	0.7	0.7
Silence Zone 8	73.3	146.6	75.4	150.8	74.6	149.2	0.7	0.7	0.7
Silence Zone 9	75.4	150.8	76.2	152.4	76.3	152.6	0.7	0.7	0.7
Silence Zone 10	80.2	160.4	80.7	161.4	80.9	161.8	0.8	0.8	0.8

**Table 4:** Noise monitoring data and comparison of noise levels with CPCB and NEI for T1, T2, and T3 time slots in industrial, residential zones, and traffic junction study sites in urban Puducherry, South India, 2021 LAeq: Hourly A-weighted equivalent sound level. Central Pollution Control Board (CPCB): %. NEI: Noise Exposure Index.

Study Sites	LAeq	CPCB %	LAeq	CPCB %	LAeq	CPCB %	NEI		
	T1		T2		T3		T1	T2	T3
Industrial Zone 1	72.8	97.1	73.1	97.5	73.2	97.6	0.7	0.7	0.7
Industrial Zone 2	67.8	90.4	65.0	86.7	68.6	91.5	0.6	0.6	0.7
Industrial Zone 3	69.3	92.4	67.8	90.4	71.6	95.5	0.7	0.6	0.7
Industrial Zone 4	78.8	105.1	77.9	103.9	76.7	102.3	0.8	0.7	0.7
Residential Zone 1	69.7	126.7	69.6	126.5	73.1	132.9	0.7	0.7	0.7
Residential Zone 2	73.4	133.5	70.7	128.5	68.4	124.4	0.7	0.7	0.7
Residential Zone 3	72.0	130.9	70.8	128.7	71.2	129.5	0.7	0.7	0.7
Residential Zone 4	79.0	143.6	79.6	144.7	80.7	146.7	0.8	0.8	0.8
Residential Zone 5	74.4	135.3	70.8	128.7	70.1	127.5	0.7	0.7	0.7
Residential Zone 6	73.9	134.4	74.8	136.0	76.1	138.4	0.7	0.7	0.7
Traffic Junction 1	80.1	123.2	81.3	125.1	82.6	127.1	0.8	0.8	0.8
Traffic Junction 2	77.2	118.8	78.4	120.6	78.1	120.2	0.7	0.7	0.7
Traffic Junction 3	80.3	123.5	79.1	121.7	81.2	124.9	0.8	0.8	0.8
Traffic Junction 4	79.8	122.8	80.8	124.3	81.2	124.9	0.8	0.8	0.8
Traffic Junction 5	78.1	120.2	77.0	118.5	77.6	119.4	0.7	0.7	0.7
Traffic Junction 6	80.1	123.2	78.5	120.8	78.8	121.2	0.8	0.7	0.8

In commercial zones, noise level LAeq varies from 67.5 dB to 81.0 dB; the highest recorded noise level was 81.0 dB, and the lowest recorded noise level was 67.5 dB (124.6% and 103.8% above the CPCB limit). In silence zones, noise level LAeq varies from 69.4 dB to 83.1 dB. The highest recorded noise level was 83.1 dB, and the lowest recorded noise level was 69.4 dB (166.2% and 138.8% above the CPCB limit). In the industrial zone, the noise level varies from 65.0 dB to 78.8 dB; the highest recorded noise level was 78.8 dB, and the lowest recorded noise level was 65.0 dB (105.1% above the CPCB limit and 13.3% below the CPCB limit). In residential zones, the noise level (LAeq) varies from 68.4 dB to 80.7 dB. The highest noise level recorded was 80.7 dB, and the lowest noise level recorded was 68.4 dB (146.7% and 124.4% above the CPCB limit). In traffic intersections, the noise level (LAeq) varies from 77.0 dB to 82.6 dB. The highest noise level recorded was 82.6 dB, and the lowest noise level was recorded at 77.0 dB (127.1% and 118.5% above the CPCB limit).

In the T1, T2, and T3 time slots, the highest noise level LAeq was recorded in the silence zone illustrated in Table 3, where the noise level varies from 69.4 dB to 83.1 dB. Silence zone study sites recorded the highest decibel fluctuating from 82.0 dB to 83.1 dB (164% to 166% above the CPCB limit), and the lowest noise level LAeq was recorded in the industrial zone illustrated in Table 3, where the noise level varies from 65.0 dB to 68.6 dB (86.7% to 91.5% below the CPCB limit).

The NEI index is directly proportional to LAeq; having a high LAeq also has a similar trend in NEI that has been observed in 2021, and it varies between 0.6 and 0.8 dB. It is also a measure for noise-induced hearing loss. NEI >1 was considered exposure to excessive noise levels greater than the prescribed limit. Table 3 illustrates the highest NEI recorded in the silence zone study site for T1, T2, and T3 time slots due to heavy traffic during peak hours and the lowest NEI recorded in all three-time slots in the industrial zone study site illustrated in Table 4. 

Tables 5, 6 illustrate that the Traffic Noise Index (TNI) was measured for commercial, silence, industrial, residential zones, and traffic intersections in T1, T2, and T3 time slots.

**Table 5:** Noise indices (TNI, NC, and LNP) for T1, T2, and T3 time slots in commercial and silence zones study sites in urban Puducherry, South India, 2021 TNI: Traffic Noise Index; NC: Noise Climate; Lnp: Noise Pollution Level

Study Sites	TNI			NC			Lnp		
	T1	T2	T3	T1	T2	T3	T1	T2	T3
Commercial Zone 1	64.5	60.8	66.9	4.9	3.9	5.1	82.3	80.8	84.5
Commercial Zone 2	95.7	95.7	95.7	15.3	15.3	15.3	89.1	89.1	89.1
Commercial Zone 3	72.8	67.3	66.4	7.0	5.1	4.7	86.2	84.4	84.9
Commercial Zone 4	61.3	81.0	96.1	9.5	13.2	17.9	68.0	80.4	85.2
Commercial Zone 5	98.8	128.6	94.7	15.5	22.0	15.1	96.3	105.8	89.0
Commercial Zone 6	81.3	152.2	72.8	11.3	31.2	9.5	85.3	118.3	82.8
Commercial Zone 7	73.1	68.2	67.6	8.3	7.2	6.1	83.7	80.7	82.9
Commercial Zone 8	70.0	69.7	84.1	8.2	8.0	11.7	80.0	80.7	84.9
Commercial Zone 9	84.7	84.0	82.8	10.6	10.7	10.2	88.3	87.8	87.3
Commercial Zone10	85.7	80.6	69.1	10.8	9.9	5.6	89.3	86.4	85.6
Silence Zone 1	99.9	76.5	73.2	16.2	10.0	8.9	93.4	81.7	81.0
Silence Zone 2	89.4	76.9	78.4	12.5	8.9	9.6	91.2	84.2	84.4
Silence Zone 3	81.0	101.8	96.1	13.2	19.3	17.9	80.4	90.0	85.2
Silence Zone 4	93.2	89.4	93.6	12.7	11.7	12.8	92.6	91.1	92.9
Silence Zone 5	68.4	63.5	67.8	5.9	4.7	5.9	83.7	81.6	83.4
Silence Zone 6	63.2	62.8	64.1	5.7	5.6	5.7	78.3	80.1	80.7
Silence Zone 7	63.9	59.9	64.8	6.4	5.8	7.4	78.4	76.0	76.0
Silence Zone 8	70.1	64.0	63.4	8.0	5.9	5.7	80.6	79.7	79.5
Silence Zone 9	93.0	102.4	113.6	14.6	16.0	19.3	91.4	95.0	100.4
Silence Zone 10	86.2	85.1	83.8	11.0	10.6	10.1	89.7	89.6	89.2

**Table 6:** Noise indices (TNI, NC, and LNP) for T1, T2, and T3 time slots in industrial, residential zones, and traffic junction study sites of urban Puducherry, South India, 2021 TNI: Traffic Noise Index; NC: Noise Climate; Lnp: Noise Pollution Level

Study Sites	TNI			NC			Lnp		
	T1	T2	T3	T1	T2	T3	T1	T2	T3
Industrial Zone 1	101.1	94.4	168.5	18.7	16.7	35.2	96.3	92.8	128.0
Industrial Zone 2	76.3	65.7	85.8	11.5	9.1	14.5	78.6	72.5	80.3
Industrial Zone 3	75.3	107.1	97.4	10.8	20.9	17.2	79.5	93.9	95.0
Industrial Zone 4	78.5	79.9	60.0	9.5	10.0	4.1	85.1	85.5	80.1
Residential Zone 1	96.9	77.4	99.0	19.2	11.7	14.7	85.9	80.3	90.2
Residential Zone 2	54.1	79.5	62.5	3.2	11.7	7.4	76.5	80.7	74.9
Residential Zone 3	101.8	101.8	96.1	19.3	19.3	17.9	90.0	90.0	85.2
Residential Zone 4	85.5	83.9	84.7	11.2	10.4	10.5	88.2	88.6	89.0
Residential Zone 5	74.0	74.8	79.8	9.0	11.1	12.6	82.9	78.4	80.5
Residential Zone 6	82.9	120.6	80.3	12.6	24.4	11.0	83.8	100.3	84.9
Traffic Junction 1	76.8	77.9	92.0	8.6	8.9	12.5	85.9	85.9	91.8
Traffic Junction 2	85.6	99.2	118.1	12.4	14.1	19.6	91.3	95.3	103.1
Traffic Junction 3	105.4	68.8	72.0	16.4	5.9	6.0	96.6	84.7	86.9
Traffic Junction 4	82.4	80.7	80.9	10.1	9.4	9.7	87.5	87.2	87.0
Traffic Junction 5	64.3	60.4	76.9	4.8	3.9	9.0	82.5	80.6	85.6
Traffic Junction 6	81.8	80.6	79.5	10.0	9.9	9.5	87.3	86.1	86.2

The highest TNI was observed in traffic junction (80.3 dB [A]), commercial (152.2 dB [A]), and industrial (168.5 dB [A]) zones study sites due to the high fluctuation of noise climate (NC), 16.4 dB [A]), 31.2 dB (A), and 35.2 dB (A), respectively, in study sites. The lowest TNI was recorded in residential (73.4 dB [A]), silence (59.9 dB [A]), and industrial (60.0 dB [A]) zones study sites, which may have resulted from low NC of 3.2 dB (A), 5.8 dB (A), and 4.1 dB (A), respectively, slow traffic flow, and the presence of additional noise-producing sources.

In T1, T2, and T3 time slots, Tables 5, 6 illustrate how noise pollution level (Lnp) was measured for commercial, silence, industrial residential zones, and traffic intersections. The maximum Lnp was observed in traffic junctions at 96.6 dB (A), commercial 118.3 dB (A), and industrial 128.0 dB (A) zones study sites, while the lowest was found in commercial 68.0 dB (A), industrial 72.5 dB (A), and residential 74.9 dB (A) zones study sites.

## Discussion

The CPCB classification was employed for site selection in the current study, which was carried out in July and August 2021. Thirty-six locations were selected from Puducherry's commercial, residential, silence, and traffic junction zones. Urban Puducherry is rising and becoming more crowded, which may be the reason that noise levels vary greatly between sites, gradually escalating or decreasing depending on location. The study from Philadelphia also shows the neighborhood's substantial temporal variability in noise levels. [18] In addition, the Mumbai Metropolitan Region found that all 34 study sites for silence zones in nine cities exceeded CPCB standards both during the day and at night. This was exacerbated by unplanned development, indicating that most silence zones are surrounded by residential and commercial areas [6]. Peshawar's study's findings, which also show noticeable seasonal fluctuations in noise levels in the commercial zone, are consistent with ours [19]. Due to the significant volume of traffic in the afternoon compared to the morning and evening, an Assam study found that the noise level in commercial areas was in the 80 dB to 90 dB range. [20], and commercial zones exceeded the CPCB limit reported by the Mumbai study [6]. According to a study conducted in the Mumbai Metropolitan Area, the industrial zone has less noise than other zones, and within the industrial zone, the areas that are most distant from the city have lower noise limits than all other industrial locations [6]. The facts were also reported in West Bengal [21] and Assam studies [20] and corroborated our study findings. Both studies attribute a large portion of the noise pollution to the traffic flow on the road network; for the West Bengal study, daytime residential noise levels varied from 53 dB to 76 dB, and for the Assam study, it was 65 dB to 75 dB [20,21].

A study conducted in urban Puducherry in 2015 revealed that the noise level in all the study sites (except industrial zones), including silence, residential, commercial zones, and traffic junctions, exceeded the CPCB daytime limit, and out of that, 17% of the study sites had a noise level (LAeq) ranging from 80 dB to 90 dB [15].

Imphal city noise assessment also shows comparable results with our study, where NEI was greater than one at multiple time slots due to heavy traffic in adjacent areas [22].

TNI is a measure to estimate traffic annoyance; hence, the variation in the traffic flow will affect the TNI. Gorakhpur [17] and Varanasi [8] point out the same: due to heavy traffic on the highway, they observed the high TNI values. A similar result for noise pollution level (Lnp) can be seen in Gorakhpur [17] and Varanasi studies [8], and their results are comparable with our findings.

We have several strengths and limitations in this study. We used the exact study site locations from the 2015 noise survey and to assess the current noise level and use of an advanced SLM. There are a few limitations to this research, foremost among them that some study sites were inaccessible because of Puducherry's urban land use and cover patterns and restrictions on the movement of vehicles during the COVID-19 pandemic.

Government should facilitate the development/reinforcement of noise-control rules and policies, and this study's results will help to develop a data repository for urban planning. Plantations of greenery close to the road can facilitate absorbing the noise and thus minimize the noise levels. The school curriculum might include the detrimental health consequences of noise pollution so that children can learn early in life.

## Conclusions

This study revealed the extent and depth of the noise pollution levels in urban Puducherry. The noise assessment at selected sites in urban Puducherry shows that the majority of the study sites are well above the CPCB's daytime standards, highlighting an urgent need to curb noise levels. The findings revealed that the rise in noise pollution levels can be attributed to the growing number of vehicles and transportation systems. It is imperative to implement effective measures to lower the noise levels and ensure the health and well-being of the residents.
